# The Xiphoid Variation: A Bifid Perspective

**DOI:** 10.51894/001c.144660

**Published:** 2025-09-30

**Authors:** Rory White

**Affiliations:** 1 College of Osteopathic Medicine Michigan State University

## 132

### INTRODUCTION

The xiphoid process is the inferior end and smallest portion of the sternum, partially forming the anterior wall of the thorax and the inferior thoracic aperture, making it crucial in clinical practice. The xiphoid process begins as a cartilaginous structure that ossifies during adulthood and is typically singular. It contains a demi-facet bilaterally where the end of each seventh costal cartilage articulates. Although variations such as a bifid (split) and trifid xiphoid process are relatively rare, when they do occur, it is typically found in males. These variations can have implications for clinical procedures, trauma management, and anatomical assessments. This case study describes the identification of a bifid xiphoid process in a female cadaver, highlighting its potential relevance in clinical practice.

### MATERIALS AND METHODS

During a dissection elective course, two medical students conducted a dissection on the thoracic region of a 64-year-old female donor. The dissection followed a protocol adapted from Grant’s Dissector which focused on identifying thoracic structures, including the sternum, and xiphoid process. Upon removing the chest plate, the xiphoid was identified and noted to have a bifid characteristic.

### RESULTS

The xiphoid process sprouted into two bony segments at its distal end, each extending laterally. The bifurcation was evident, with the two portions of the xiphoid connected to the body of the sternum through a flexible, cartilaginous junction. The bifid division occurred approximately 2.5 cm from the tip of the process, and interestingly, one was found to be in line with the costal margin, making it difficult to palpate or identify from an anterior position.

### CONCLUSION

The abnormal findings of this variant when performing procedures involving the chest, such as CPR, abdominal surgery, or thoracic imaging, should be known to clinicians, as it could influence the location of certain anatomical structures. Additionally, the bifid xiphoid may present challenges in trauma management or when interpreting radiological images of the sternum as it can mimic an epigastric mass and cause pain. Further studies to assess the prevalence and clinical significance of bifid xiphoid processes in the general population would be valuable in enhancing surgical precision and patient care.

